# Polymer-derived SiOC reinforced with core–shell nanophase structure of ZrB_2_/ZrO_2_ for excellent and stable high-temperature microwave absorption (up to 900 °C)

**DOI:** 10.1038/s41598-023-27541-3

**Published:** 2023-01-06

**Authors:** Yujun Jia, Ni Yang, Shaofan Xu, Alexander D. Snyder, Jason F. Patrick, Rajan Kumar, Dajie Zhang, Chengying Xu

**Affiliations:** 1 Department of Mechanical and Aerospace Engineering, North Carolina State University, Raleigh, NC 27695 USA; 2 State Key Laboratory of Solidification Processing, Northwestern Polytechnical University, Xi’an, 710072 People’s Republic of China; 3 FAMU-FSU College of Engineering, Florida State University, Tallahassee, FL 32310 USA; 4 Department of Materaials Science and Engineering, The Johns Hopkins University, 3400 North Charles Street, Baltimore, MD 21218 USA; 5 Research and Exploratory Development Department, The Johns Hopkins Applied Physics Laboratory, 11100 Johns Hopkins Road, Laurel, MD 20723 USA; 6 Department of Civil, Construction, and Environmental Engineering, North Carolina State University, Raleigh, NC 27695 USA

**Keywords:** Materials science, Ceramics, Composites

## Abstract

Microwave absorbing materials for high-temperature harsh environments are highly desirable for aerodynamically heated parts and engine combustion induced hot spots of aircrafts. This study reports ceramic composites with excellent and stable high-temperature microwave absorption in air, which are made of polymer-derived SiOC reinforced with core–shell nanophase structure of ZrB_2_/ZrO_2_. The fabricated ceramic composites have a crystallized t-ZrO_2_ interface between ZrB_2_ and SiOC domains. The ceramic composites exhibit stable dielectric properties, which are relatively insensitive to temperature change from room temperature to 900 °C. The return loss exceeds − 10 dB, especially between 28 and 40 GHz, at the elevated temperatures. The stable high-temperature electromagnetic (EM) absorption properties are attributed to the stable dielectric and electrical properties induced by the core–shell nanophase structure of ZrB_2_/ZrO_2_. Crystallized t-ZrO_2_ serve as nanoscale dielectric interfaces between ZrB_2_ and SiOC, which are favorable for EM wave introduction for enhancing polarization loss and absorption. Existence of t-ZrO_2_ interface also changes the temperature-dependent DC conductivity of ZrB_2_/SiOC ceramic composites when compared to that of ZrB_2_ and SiOC alone. Experimental results from thermomechanical, jet flow, thermal shock, and water vapor tests demonstrate that the developed ceramic composites have high stability in harsh environments, and can be used as high-temperature wide-band microwave absorbing structural materials.

## Introduction

High-temperature microwave absorbing materials are of great interest for aerodynamically heated parts of supersonic and hypersonic systems, such as head cone, engine inlet and exhaust nozzle, and aeroshells. These materials are used for the dissipation of the electromagnetic (EM) wave to reduce radar signature^1–3^. The above applications not only require the materials to resist oxidation but also maintain good microwave absorption at high temperatures. Because of their relatively low density and good high-temperature resistance, ceramic materials are considered to be the most suitable materials for such applications. Currently, microwave absorbing ceramics include oxide-based ceramics and non-oxide based ceramics (SiC, SiCN, and Si_3_N_4_) via polymer-derived routes. For example, SiC/SiO_2_ composites showed an effective absorption bandwidth (EAB, < − 10 dB) of 4.2 GHz at a thickness of 2.8 mm at 500 °C in the X band^4^. C_f_/SiCNFs/Si_3_N_4_ composites had a return loss (*RL*) as low as − 20.3 dB at 800 °C for a thickness of 2 mm^5^. The EAB of SiC_f_/SiC composites is 2.8 GHz at a thickness of 2.5 mm at 600 °C for the X band^6^.

Among these ceramics or ceramic composites, polymer-derived ceramics (PDCs) are considered to be promising high-temperature EM absorption ceramics due to their tunable electrical and dielectric properties as well as relatively low processing temperature, excellent oxidation resistance at high temperature, and flexibility in design and manufacturing^7–16^. The average reflectivity of polymer-derived SiC is ~ − 9.9 dB due to the formation of nanocrystalline SiC and the free carbon nanodomains. In order to further improve the microwave absorption of the PDCs, electrically conductive phases were incorporated into the matrix to improve conduction loss. For example, after the addition of MWCNT, the minimum *RL* of PDC-SiBCN reaches − 32 dB with an EAB of 3 GHz in X-band, showing a better wave-absorbing ability than SiBCN treated at the same temperature^17^. The minimal *RL* of SiC/SiOC ceramics reached − 61 dB at 8.6 GHz and the widest EAB reached 3.5 GHz in the X-band^18^.

For even higher temperature applications, electrically conductive ultra-high-temperature ceramics (UHTCs), such as HfC and ZrB_2_, were introduced into the PDCs, because these ceramics have not only excellent electrical conductivity, but also super high melting points, high-temperature mechanical property retention, excellent corrosion resistance, and good oxidation resistance at high temperatures. For example, the EAB of polymer-derived (SiC/HfC/C)/SiO_2_ composites covers 72% of the X band at a thickness of 3.33 mm^14^. The EAB of polymer-derived SiOC-ZrB_2_ composites covers the entire Ka band at a thickness of 3 mm at room temperature (*RT*)^19^.

For PDCs, the more addition of the UHTCs, the higher the electrical conductivity of the composites can be obtained. Unfortunately, high conductivity also results in significant interfacial impedance mismatching between the UHTCs and PDCs. Consequently, reflection increases when the UHTC conduction network is formed in the PDCs, especially at high temperatures, which deteriorated the absorption ability of the entire materials. To address the above challenge and achieve high-efficiency EMW absorption at high temperatures, both strong absorption ability and impedance matching are required. Therefore, it is necessary to design the microstructure of the UHTCs to improve the interfacial impedance matching ability as well as maintain the efficient loss ability at high temperatures. Herein, a dielectric oxide phase was constructed between the individual UHTC particles, and between the UHTC phase and the PDCs matrix, to form a composite nanophase for improving the high-temperature EMW absorption ability of the ceramic materials. The fabricated ceramic composites exhibited excellent EMW absorption at temperatures up to 1000 °C, revealing extraordinarily stable absorption capability. Such material systems demonstrate application feasibilities in a high-temperature harsh environment based on the thermal structural stability tests. This work provides a novel approach for adjusting the high-temperature electrical properties and achieving high-temperature microwave-absorption performance of pre-ceramic polymer derived materials.

## Results

### Thermal structure stability analysis of the ceramic composites

**Figure 1:**
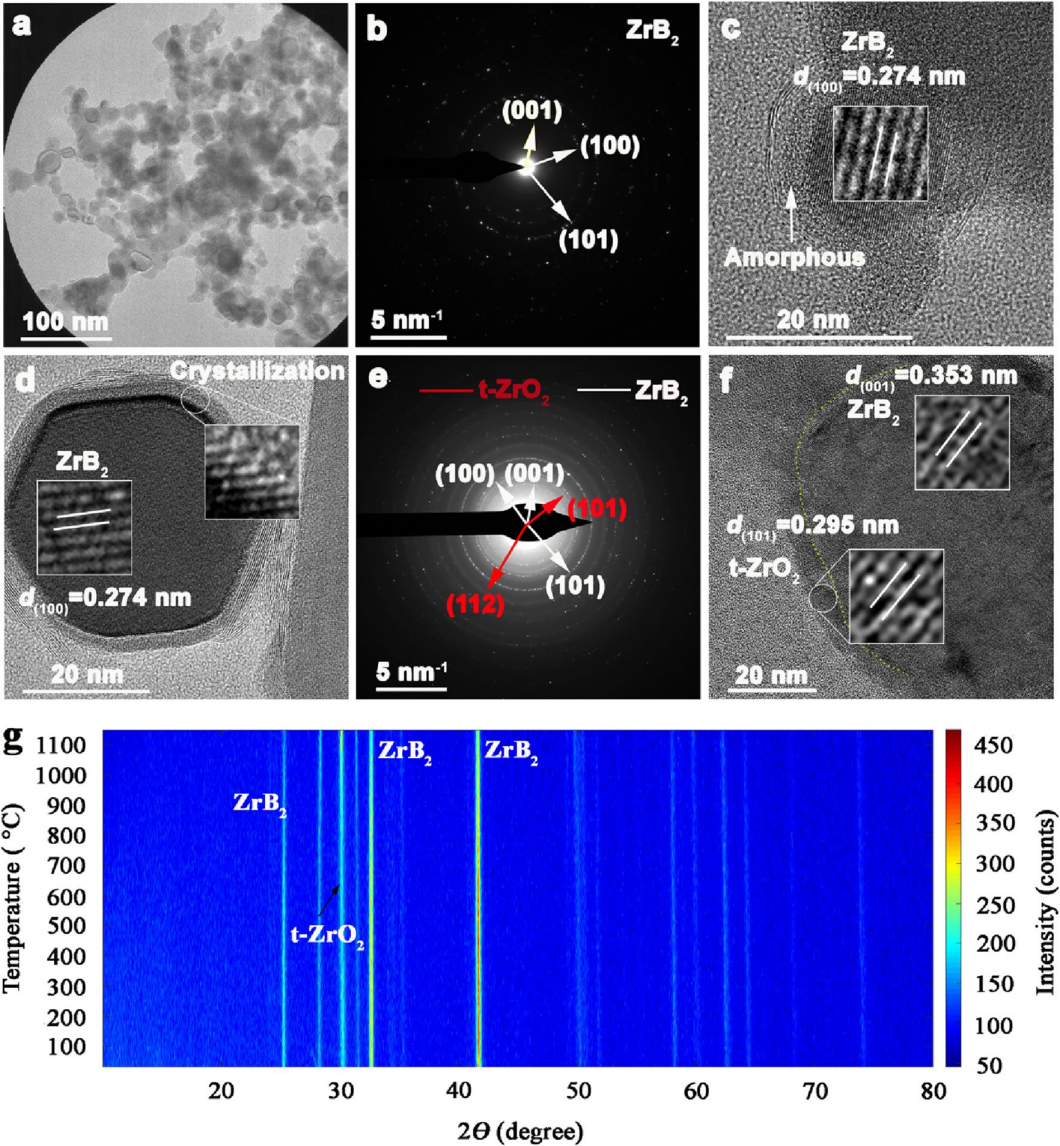
TEM analysis of the ZrB_2_ nanoparticles: (**a**) low magnification image, (**b**) SAED showing that these particles are ZrB_2_, (**c**) high magnification image revealing the nanoparticle is coated by an amorphous surface layer, (**d**) TEM image of the heat-treated ZrB_2_ nanoparticles showing crystallization of the amorphous surface. TEM analysis of the ceramic composites: (**e**) SAED showing the ceramic composites are composed of ZrB_2_, t-ZrO_2,_ and amorphous SiOC, (**f**) TEM image of the ceramic composites revealing a crystallized t-ZrO_2_ interface between the ZrB_2_ nanoparticles and SiOC matrix, (**g**) In-situ XRD characterizations from the ceramic composites at 25–1150 °C in air gas, with the ramp rate of 10 °C/min.

Understanding the properties of ZrB_2_ nanoparticles is important in this work. TEM analysis of the as-received ZrB_2_ nanoparticles is shown in Fig. 1a–c, revealing that the ZrB_2_ nanoparticles are coated by an amorphous surface layer, with the nanoparticle size being between 18 and 50 nm. The formation of this pre-existing layer is due to the natural oxidation in air. Under heat treatment at 1000 °C, the amorphous layer still exists while interface crystallization occurs between the amorphous layer and the ZrB_2_ nanoparticles (Fig. 1d), indicating the thermal stability of received ZrB_2_ nanoparticles. Figure 1e,f show the TEM analysis of the as-prepared ceramic composites. The selected area electron diffraction (SAED) reveals that the ceramic composites are mainly composed of ZrB_2_ and t-ZrO_2_. High resolution TEM image (Fig. 1f) shows that the SiOC matrix is amorphous and is separated from the ZrB_2_ phase by the crystallized t-ZrO_2_ interface. The electrically insulating t-ZrO_2_ interface is expected to improve the impedance matching between the ZrB_2_ filler and SiOC matrix for enhancing the introduction of the EM wave. Figure 1g shows the in-situ XRD patterns of the ceramic composites at 25–1150 °C in air with a ramp rate of 10 °C/min. It can be seen that the ceramic composites show a stable phase composition of ZrB_2_ and t-ZrO_2_ with increasing temperature. No evident phase change is observed.

**Figure 2:**
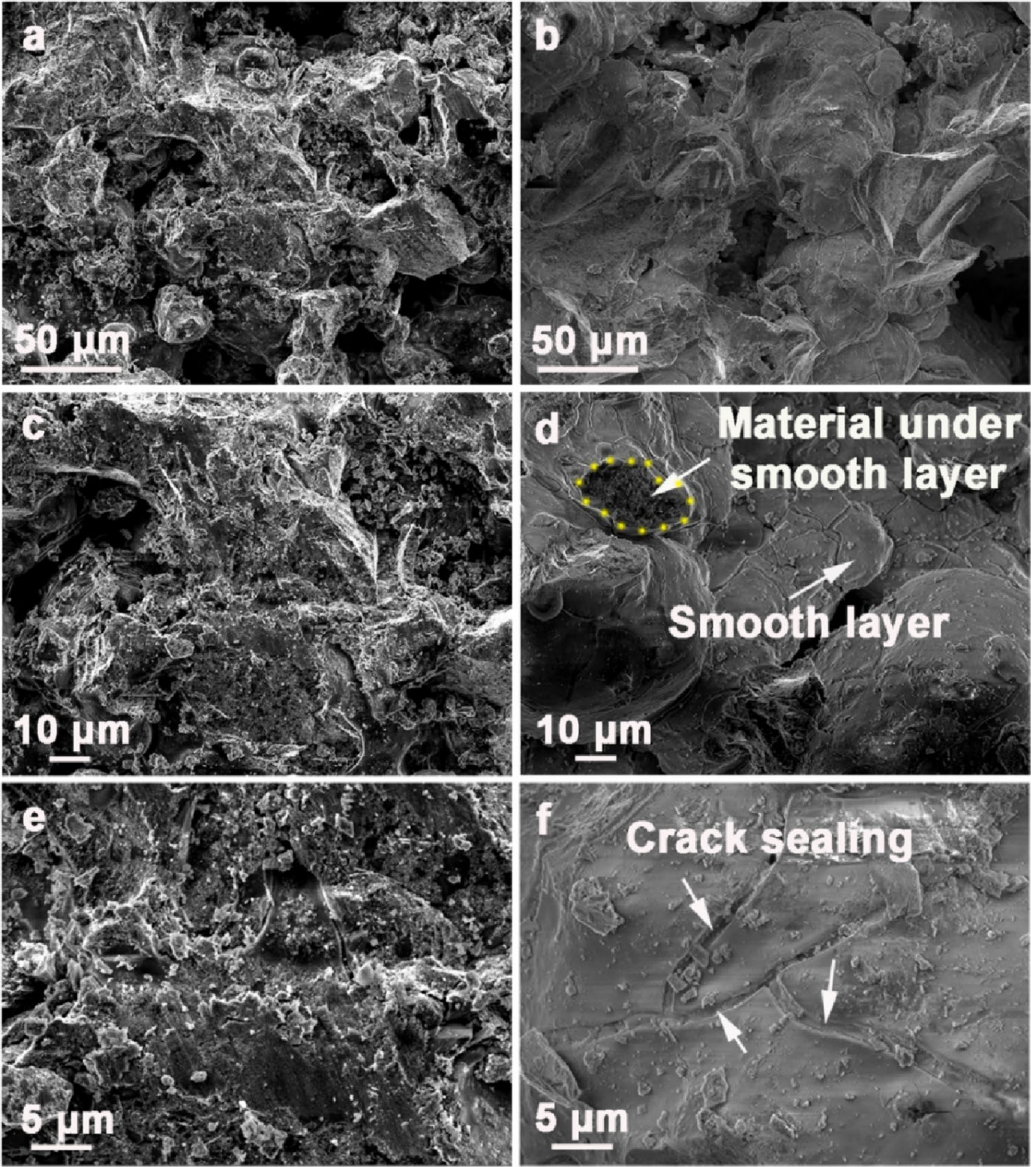
SEM images of the ceramic composites before (**a**,**c**,**e**) and after (**b**,**d**,**f**) the free space test at different magnifications.

The ceramic composites especially the ZrB_2_ nanoparticles need to survive the high-temperature harsh environment in order to maintain the capability for EM wave absorption. The surface microstructure of the ceramic composites before and after the free space test is shown in Fig. 2. The ceramic composites have uneven surface before the free space test (Fig. 2a,c,e). It is resulting from the pressing powder method during the fabrication. Cracks (Fig. 2e) can be found resulting from the fabrication process of the ceramic composites. After the free space test, the ceramic composite surface became smooth and dense (Fig. 2b,d,f). This dense oxide layer is formed due to oxidation of the surface of the ceramic composites at high temperatures, and it can serve as a protection coating impeding further oxidation. The cracks in the ceramic composites are fully sealed by the smooth layer due to their good mobility (Fig. 2f), which suggests that the ceramic composites have excellent sealing capacity and oxidation resistance at high temperatures.

**Figure 3:**
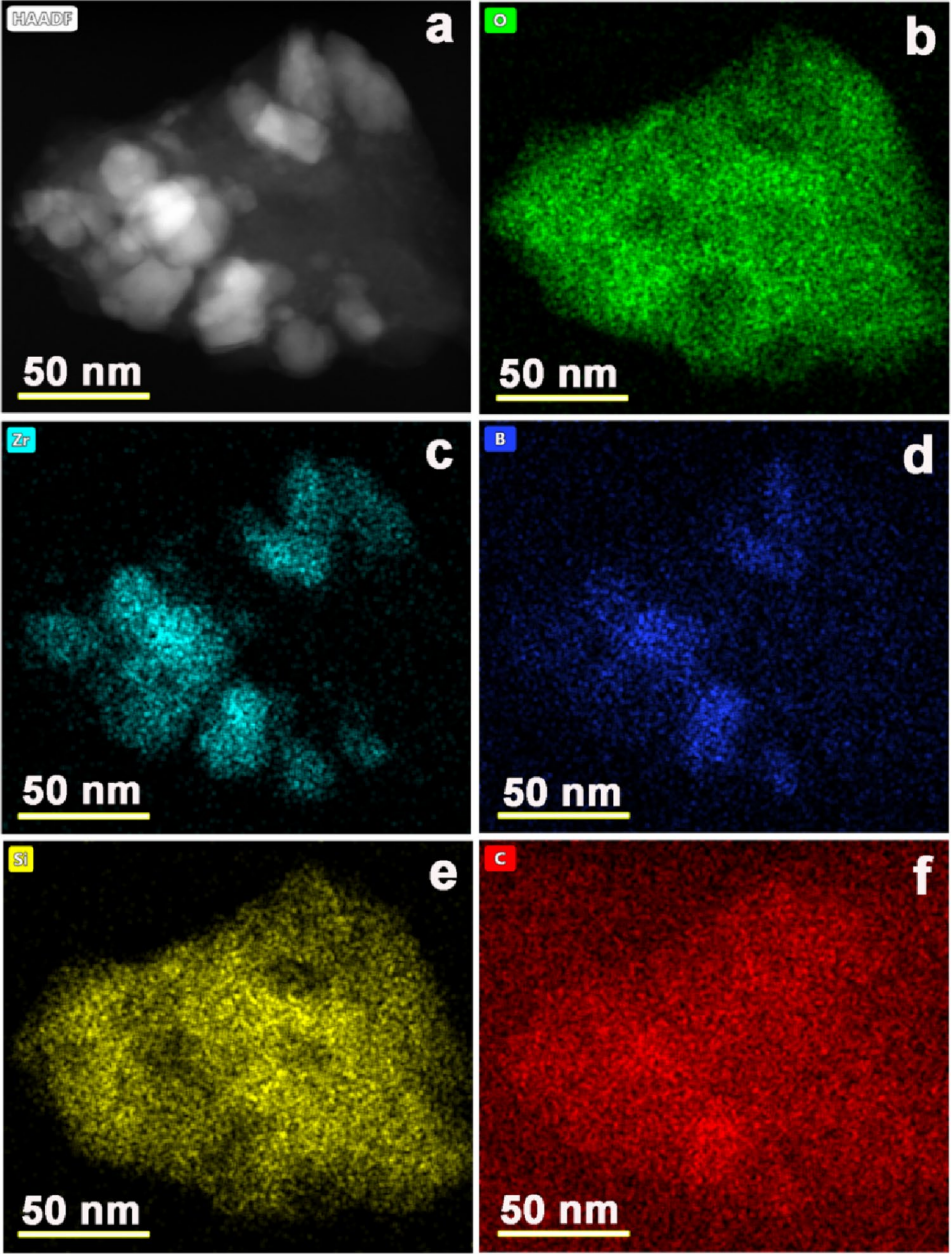
EDS analysis of the ceramic composites after the free space test. (**a**) TEM image of the analysis area. (**b**–**f**) distribution of the elements O, Zr, B, Si, and C, respectively.

Figure 3a–f shows the EDS analysis of the ceramic composites after the free space test at 1000 °C in air. It can be seen that the B element distributes uniformly inside the nanoparticles, while O exists outside of the nanoparticles. The existence of B means that ZrB_2_ survived after the high-temperature test in air, suggesting that the ceramic composites have good composition stability in a high-temperature oxidation environment.

**Figure 4:**
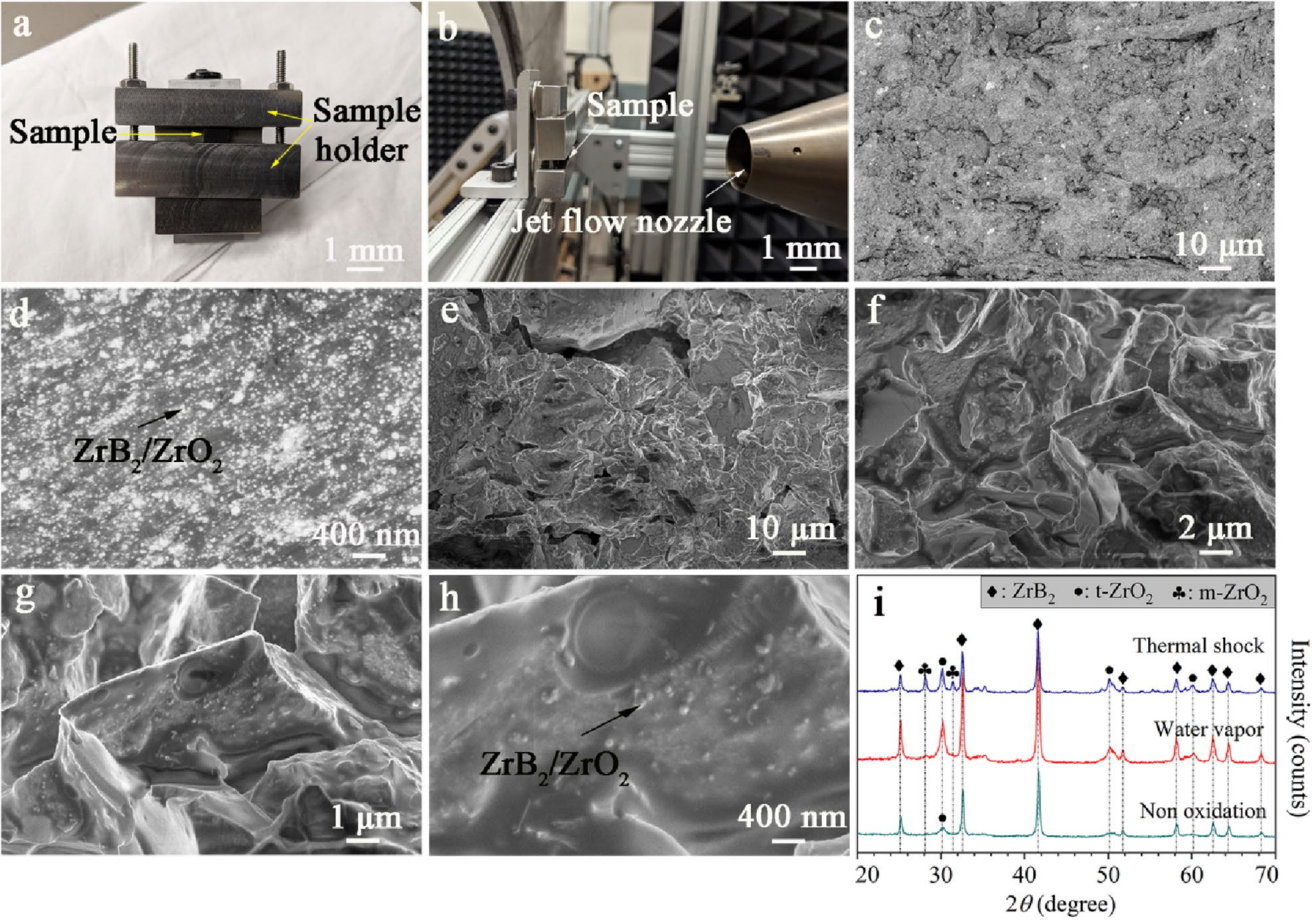
Optical images of the sample holder for the jet flow test (**a**) and supersonic (Mach 1.5) nozzle (**b**). BSE images of the ceramic composites before jet flow test (**c**,**d**). SEM images of the ceramic composites after jet flow test (**e**,**h**). XRD comparative analysis of the ceramic composites after different corrosion tests (**i**).

**Figure 5:**
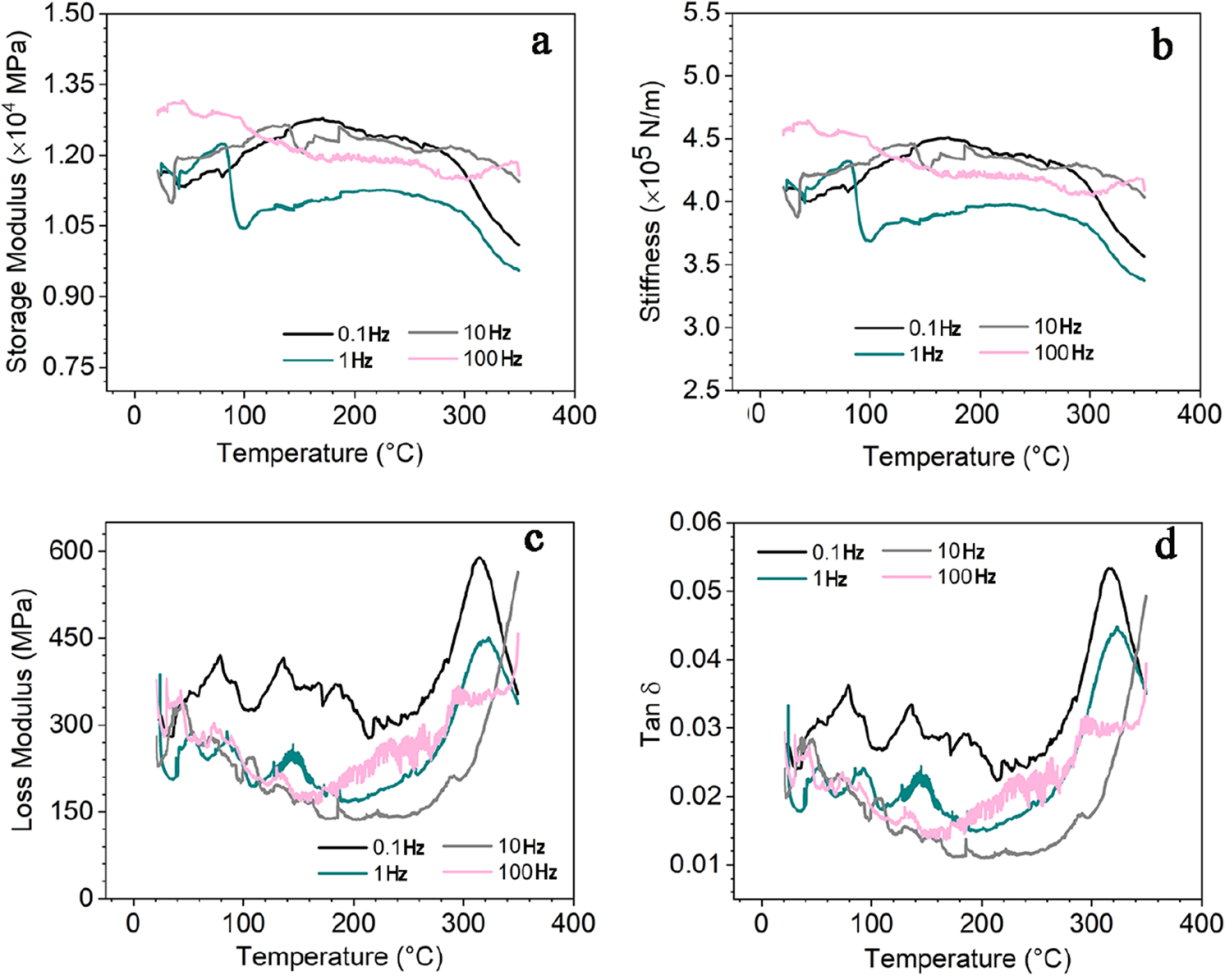
Dynamic mechanical analysis (DMA): (**a**) Storage modulus, (**b**) loss modulus, (**c**) Tan δ, and (**d**) flexural stiffness of the ceramic composites as a function of temperature and strain rate (0.1, 1.0, 10, 100 Hz).

Thermal–mechanical properties of the NL40 sample were tested under the high-velocity (Mach = 1.5) jet flow at the stagnation temperature of 254 °C. The test sample, sample holder, and the jet flow test apparatus are shown in Fig. 4a,b. Figure 4c,d is the backscattered electron image (BSE) images of the ceramic composites before the jet flow test. Structures with some porosity and homogeneous distribution of the nanoparticles in the SiOC can be observed in Fig. 5d. After the jet flow test, the pore size increased because of the blowing of the air flow (Fig. 4e). However, the nanoparticles can provide a pinning effect to prevent damage to the SiOC matrix, as seen from the SEM images in Fig. 4f–h.

To further test the corrosion resistance in harsh environment, water vapor corrosion and thermal shock test was conducted for the ceramic composites. The materials were exposed to Ar/water vapor mixture flowing (Ar: H_2_O molar ratio was of about 5:1) at 100 cm^3^/min at temperatures from *RT* to 500 °C. In such water vapor-containing environment, the primary oxidant is water vapor. The mass loss of the ceramic composites in high-temperature water vapor is 2.31 ± 0.04%, which is potentially caused by the following reactions:

1 $${\text{ZrB}}_{{2}} \left( {\text{s}} \right) \, + {\text{ 8H}}_{{2}} {\text{O}}\left( {\text{g}} \right) \, = {\text{ ZrO}}_{{2}} \left( {\text{s}} \right) \, + {\text{ 2H}}_{{3}} {\text{BO}}_{{3}} \left( {\text{g}} \right) \, + {\text{ 5H}}_{{2}} \left( {\text{g}} \right),$$

2 $${\text{C }}\left( {\text{s}} \right) + {\text{ H}}_{{2}} {\text{O}}\left( {\text{g}} \right) \, = {\text{ CO}}\left( {\text{g}} \right) \, + {\text{ H}}_{{2}} \left( {\text{g}} \right),$$

3 $${\text{SiOC}}\left( {\text{s}} \right) \, + {\text{ 2H}}_{{2}} {\text{O}}\left( {\text{g}} \right) = {\text{ SiO}}_{{2}} \left( {\text{s}} \right) \, + {\text{ CO}}\left( {\text{g}} \right) + {\text{ 2H}}_{{2}} \left( {\text{g}} \right).$$

The comparison of the XRD analysis of the sample before and after the water vapor test reveals an increased ZrO_2_ content (Fig. 4i), which agrees with the proposed reaction (1).

The thermal shock behavior of the ceramic composites was evaluated as a function of quenching temperature and quenching cycles. The ceramic specimens were heated to 800 °C in air and held at this temperature for 10 min. Then, the heated specimens were dropped by free fall into a water bath at *RT*. The XRD analysis after thermal shock (Fig. 4i) shows that the sample surface is mainly composed of ZrB_2_ and ZrO_2_. All the samples survived the water quenching thermal shock tests without cracking or breaking.

Figure 5 shows the DMA test results for the ceramic composites. The storage modulus (Fig. 5a) and stiffness (Fig. 5b) of the ceramic composites show values of (0.9–1.35) × 10^4^ MPa and (3–5) × 10^5^ N/m, respectively. The loss modulus (Fig. 5c) and tanδ (Fig. 5d) of the ceramic composites have the values of 150–600 MPa and 0.01–0.06, respectively, suggesting a low energy loss under the periodic external force environment. Therefore, the ceramic composites have good thermal environmental stability. Besides, a loss modulus/loss tangent peak appears at high temperature (300–250 °C) when the sample was applied an high frequency alternating load, revealing some damping characteristic of the ceramic composites.

### Dielectric and EM absorption properties of the ceramic composites at high temperature in air

The microwave absorption property of the ceramic composites is correlated to the complex relative permittivity. Thus, the dielectric properties of both the pure SiOC and the ceramic composites were analyzed, with EM absorption properties compared. The microwave absorption property of the ceramics is evaluated by the return loss (*RL*) calculated by Eqs. (4, 5) ^20–22^ using the complex relative permittivity based on the generalized transmission line theory and metal back plane model:

4 $$RL=20\mathrm{log} \, \left|\frac{{Z}_{\mathrm{in}}-1}{{Z}_{\mathrm{in}}+1}\right|,$$

5 $$Z_{{{\text{in}}}} = \sqrt {\frac{{\mu_{{\text{r}}} }}{{\varepsilon_{r} }}} \tanh \left[ {{\text{j}}\frac{2\pi }{c}\sqrt {\mu_{{\text{r}}} \varepsilon_{r} } fd} \right],$$

where *RL* is return loss, *c* is light velocity in vacuum, *f* is frequency, *Z*_in_ is normalized input impedance, *ε*_r_ and *μ*_r_ are relative permittivity and permeability, respectively.

**Figure 6:**
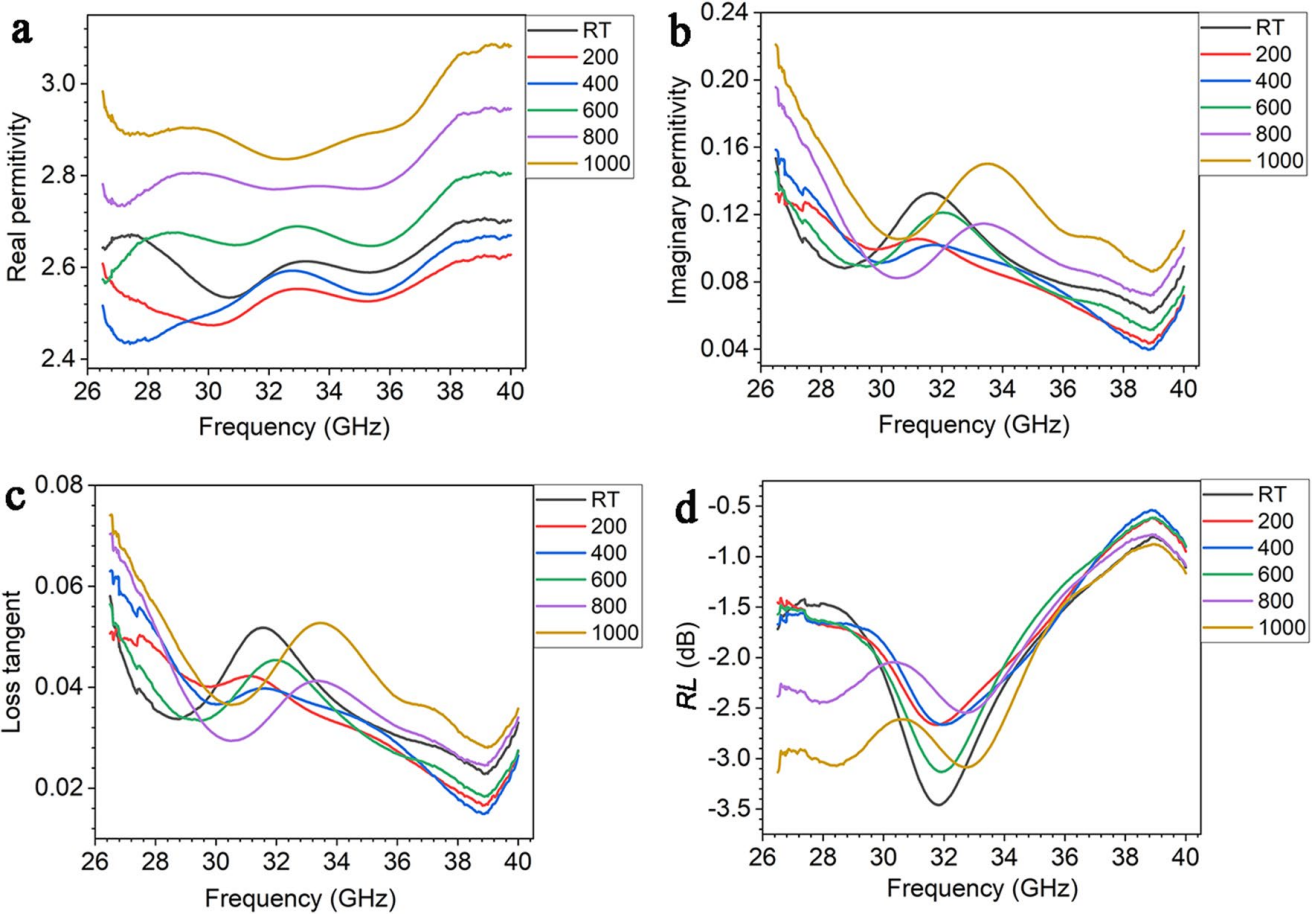
The complex permittivity (**a**,**b**), loss tangent (**c**), *RL* (**d**) of SiOC at the thickness of 4.45 mm at different temperature.

The complex permittivity of the SiOC ceramic at different temperatures is shown in Fig. 6a–c. The loss tangent values (Fig. 6c) fall into the range of about 0.015–0.075 within the Ka-band at temperatures up to 1000 °C, and the *RL* values (Fig. 6d) are between 0.60 and 3.5 dB.

**Figure 7:**
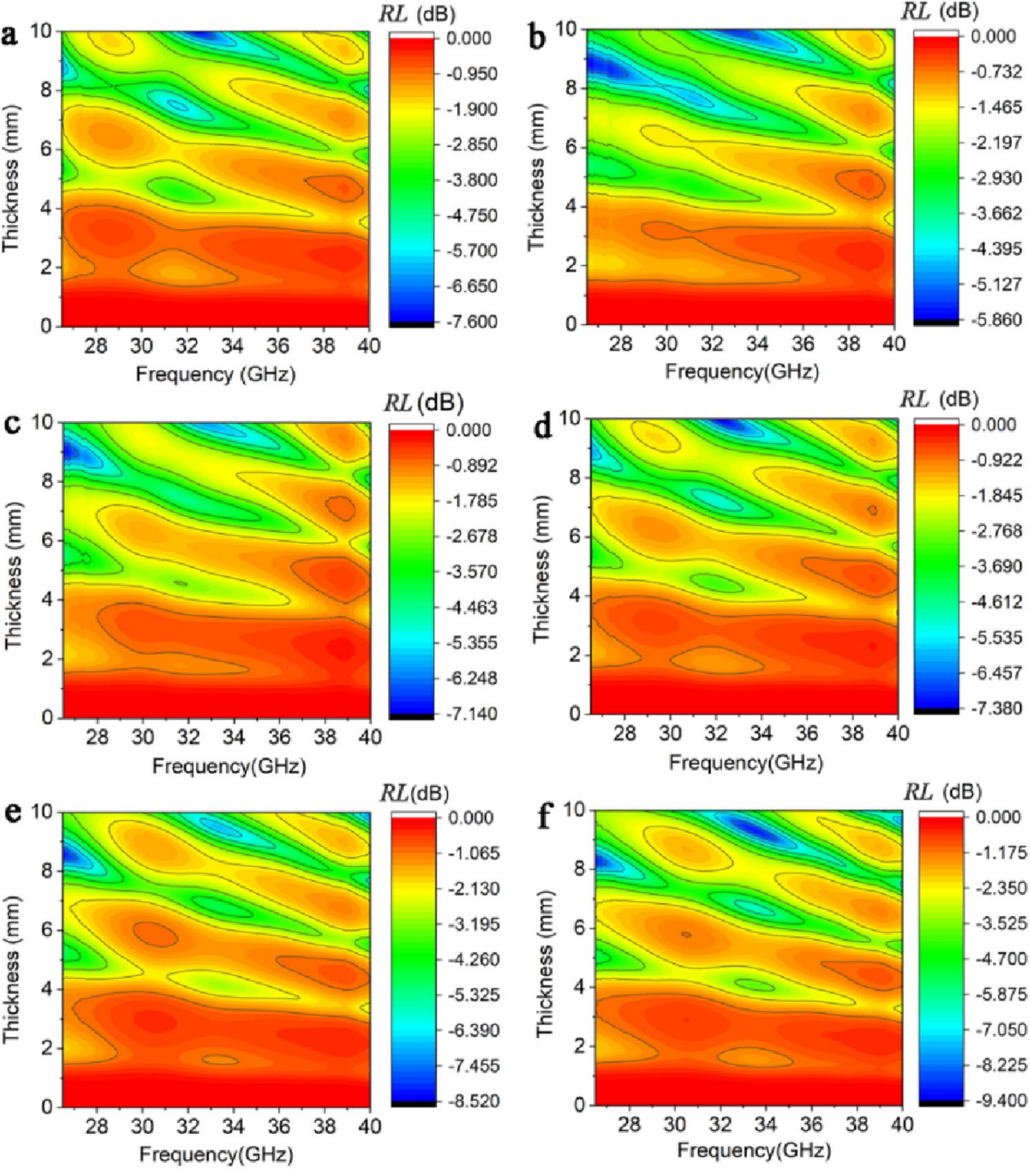
Effect of thickness on the EM absorption performance of SiOC at different temperature. (**a**) RT, (**b**) 200 °C, (**c**) 400 °C, (**d**) 600 °C, (**e**) 800 °C, (**f**) 1000 °C.

Figure 7a–f shows the effect of thickness on the EM absorption capability of SiOC at different temperatures. It is difficult to achieve *RL* values exceeding − 10 dB for the SiOC from *RT* to 1000 °C. Therefore, SiOC is not a strong EM absorbing material, and relying on SiOC alone can not achieve feasible high-temperature EM absorption coatings.

**Figure 8:**
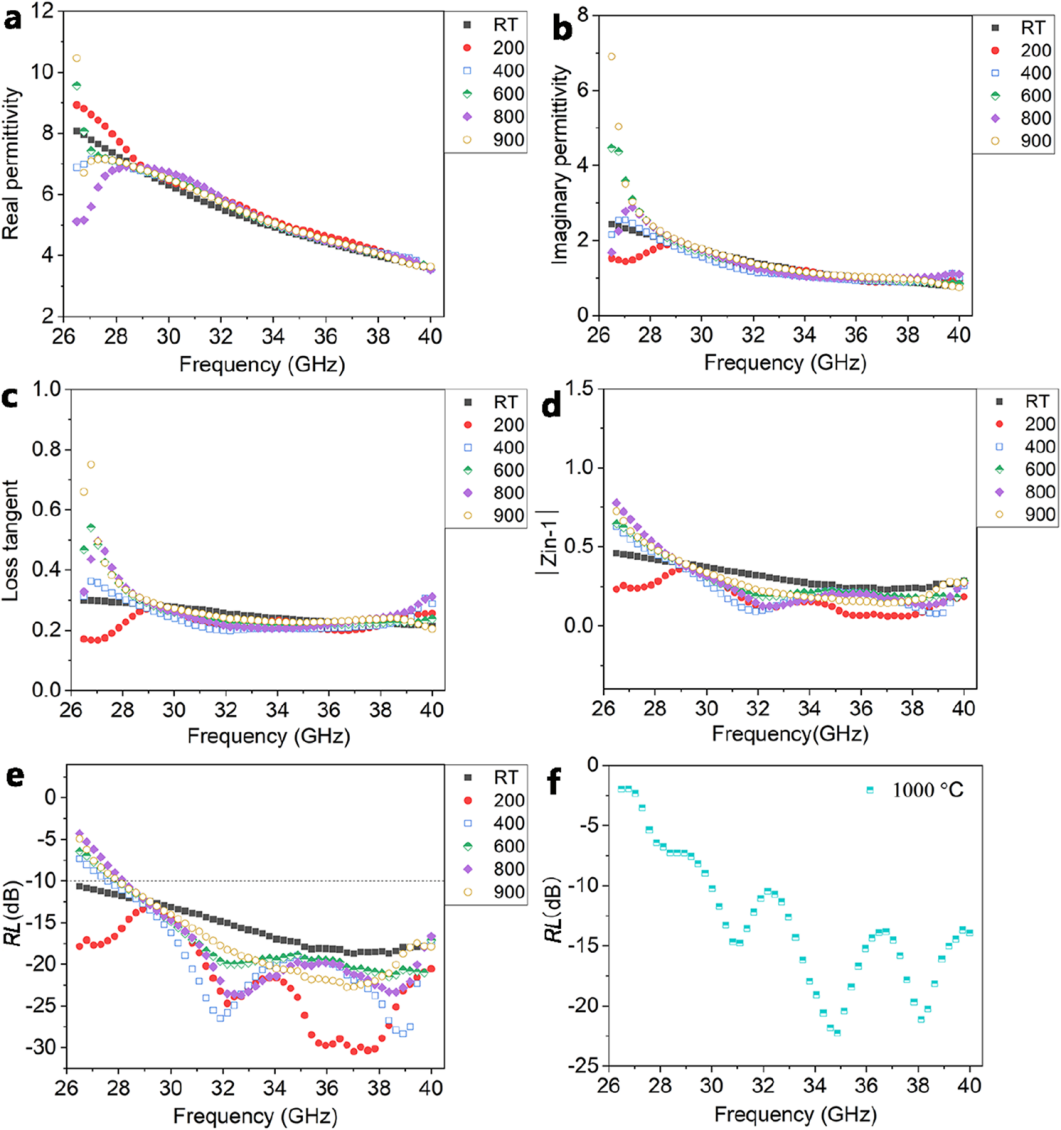
The complex permittivity (**a**,**b**), loss tangent (**c**), modulus of ($Z_{{{\text{in}}}} - 1$) (**d**) and *RL* (**e**) of the ceramic composites with a thickness of 2.9 mm at different temperatures. *RL* (**f**) of the ceramic composites with a thickness of 2.7 mm at 1000 °C.

Figure 8a–c shows the dielectric properties of the PDC SiOC/ZrB_2_/ZrO_2_ ceramic composites. The complex permittivity shows frequency dependence, but is relatively insensitive to temperature change from RT to 900 °C, especially between 28 and 40 GHz. This indicates that SiOC/ZrB_2_/ZrO_2_ composites have stable dielectric properties within a broad temperature range until 900 °C. More specific, at RT, the average real permittivity (ε′), imaginary permittivity (ε″) and loss tangent (tan δ) of the ceramic composites are 5.39, 1.40, and 0.25 in Ka-band, respectively. When the temperature increases to 400 °C, the values are 5.43, 1.33, and 0.24, respectively. At 900 °C, the corresponding values are 5.47, 1.62, and 0.28, respectively. Figure 8d shows the calculated modulus of *Z*_in_-1 (Ι*Z*_in_-1Ι) for the ceramic composites at different temperatures. The value of Ι*Z*_in_-1Ι approaching zero represents a better impedance matching with the free space. From the figure, the Ι*Z*_in_-1Ι values of the ceramic composites are similar to each other at different temperatures up to 900 °C, which indicates low reflection at the material surface. The *RL* values (Fig. 8e) of the ceramic composites with a thickness of 2.9 mm at different temperatures are calculated based on formulas (4) and (5). At such thickness, the *RL* of the ceramic composites can exceed − 10 dB at RT and 200 °C, covering the entire Ka-band. The absorption bandwidths of the ceramic composites at 400 °C, 600 °C, 800 °C and 900 °C are 12.42 GHz, 11.88 GHz, 11.61 GHz, 11.88 GHz, respectively, while that with a thickness of 2.7 mm at 1000 °C is 9.99 GHz (Fig. 8f).

When the EM wave arrives at the surface of the ceramic composites, one part of the incident EM wave can be absorbed, and the rest will be reflected on the front and back surfaces. When the thickness of material (*d*) is a quarter of the propagation wavelength (*λ*) multiplied by an odd number, it is described by Eq. (6) in Ref.^23^

6 $$d = n\lambda /4 = nc/(4f_{m} \sqrt {\left| \mu \right|\left| \varepsilon \right|} )\quad \left( {n = { 1},{ 3},{ 5}, \, \ldots } \right),$$

where *c* is light speed in vacuum, *f*_m_ is the matching frequency, *ε* is the permittivity, and *μ* is the permeability of materials. At such thicknesses, the EM wave reflected by the front surface has a phase opposite to the EM wave reflected from the back surface, leading to destructive interference and attenuation of EM wave.

**Figure 9:**
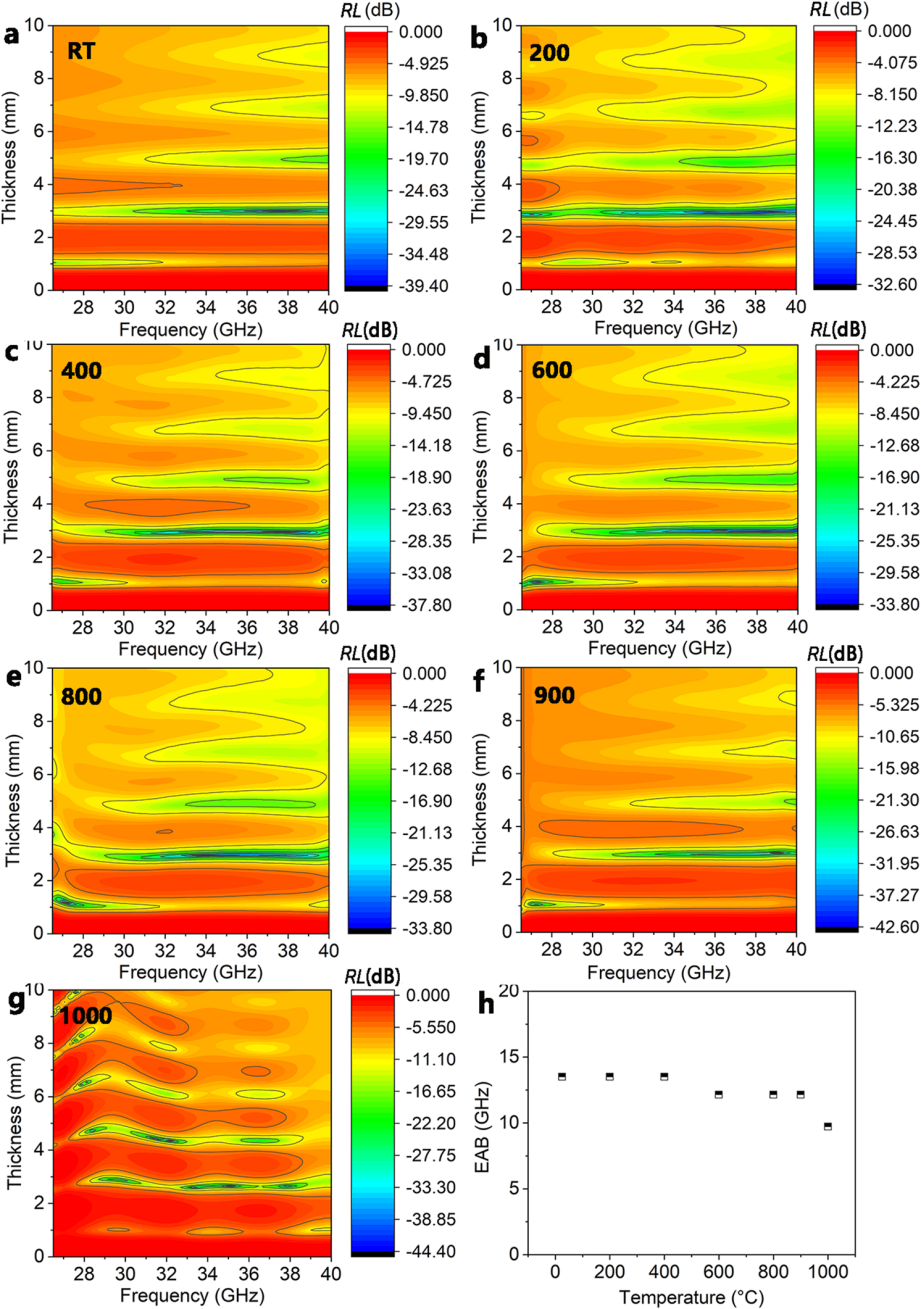
Effect of thickness on the EM absorption property of the ceramic composites at different temperatures. (**a**) RT, (**b**) 200 °C, (**c**) 400 °C, (**d**) 600 °C, (**e**) 800 °C, (**f**) 900 °C. (**h**) The EAB of the ceramic composites as a function of temperature.

Therefore, to optimize the absorption property, *RL* as a function of thickness and frequency is plotted in Fig. 9a–g. It can be seen that at the thickness range of 2–10 mm, the ceramic composites have a stable high-temperature EM wave absorption capability. The maximum absorption bandwidths of the ceramic at RT, 200 °C, and 400 °C cover the entire Ka-band. The optimized bandwidths at temperatures higher than 600 °C cover almost the entire Ka-band and stay stable with the temperature increase, as seen in Fig. 9h. At 1000 °C, the microwave absorption bandwidth of the ceramic composites is 9.99 GHz, which still covers most of the Ka-band. These features could prove that ceramic composites are suitable for high-temperature microwave absorption applications.

## Discussion

The high-temperature EM absorption property of the ceramic composites is closely related to their dielectric and electrical properties. From Fig. 6, the real permittivity of SiOC varies from 2.4 to 2.92 as the temperature increases to 1000 °C, showing a stable dielectric property. For the ceramic composites, the stable dielectric property is ascribed to the ZrB_2_/ZrO_2_ nanophases due to its dominant dielectric properties in ceramic composites. Therefore, the ZrB_2_/ZrO_2_ nanophases provide stable high-temperature dielectric property of the ceramic composites from *RT* to high temperatures.

The variation of the dielectric constant influences the impedance matching of the ceramic composites with the free space. The SiOC has low permittivity and loss tangent, which can be viewed as a wave-transparent ceramic. Thanks to the t-ZrO_2_ interface between the ZrB_2_ and SiOC, the impedance shows a transitional change and the impedance mismatching is eliminated. This allows more EM wave to be incident and facilitates the absorption by the ZrB_2_. Therefore, the ceramic composites show similar impedance values at high temperatures (Fig. 8d).

The imaginary permittivity of the ceramic composites can be described by the following Eq. (7):^24^

7 $$\varepsilon^{\prime \prime } = \frac{{\varepsilon_{s} - \varepsilon_{\infty } }}{{1 + (\omega \tau )^{2} }}\omega \tau + \frac{\sigma }{{2\pi \varepsilon_{0} f}}.$$

The first part is the polarization loss of the ceramic composites, and the second part is the conductive loss of the ceramic composites. For dielectric ceramics, the conduction loss contributes to a larger portio of the total loss, and the imaginary part can be expressed by Eq. (8) ^25^

8 $$\varepsilon^{\prime \prime } \approx \frac{\sigma }{{2\pi \varepsilon_{0} f}}.$$

**Figure 10:**
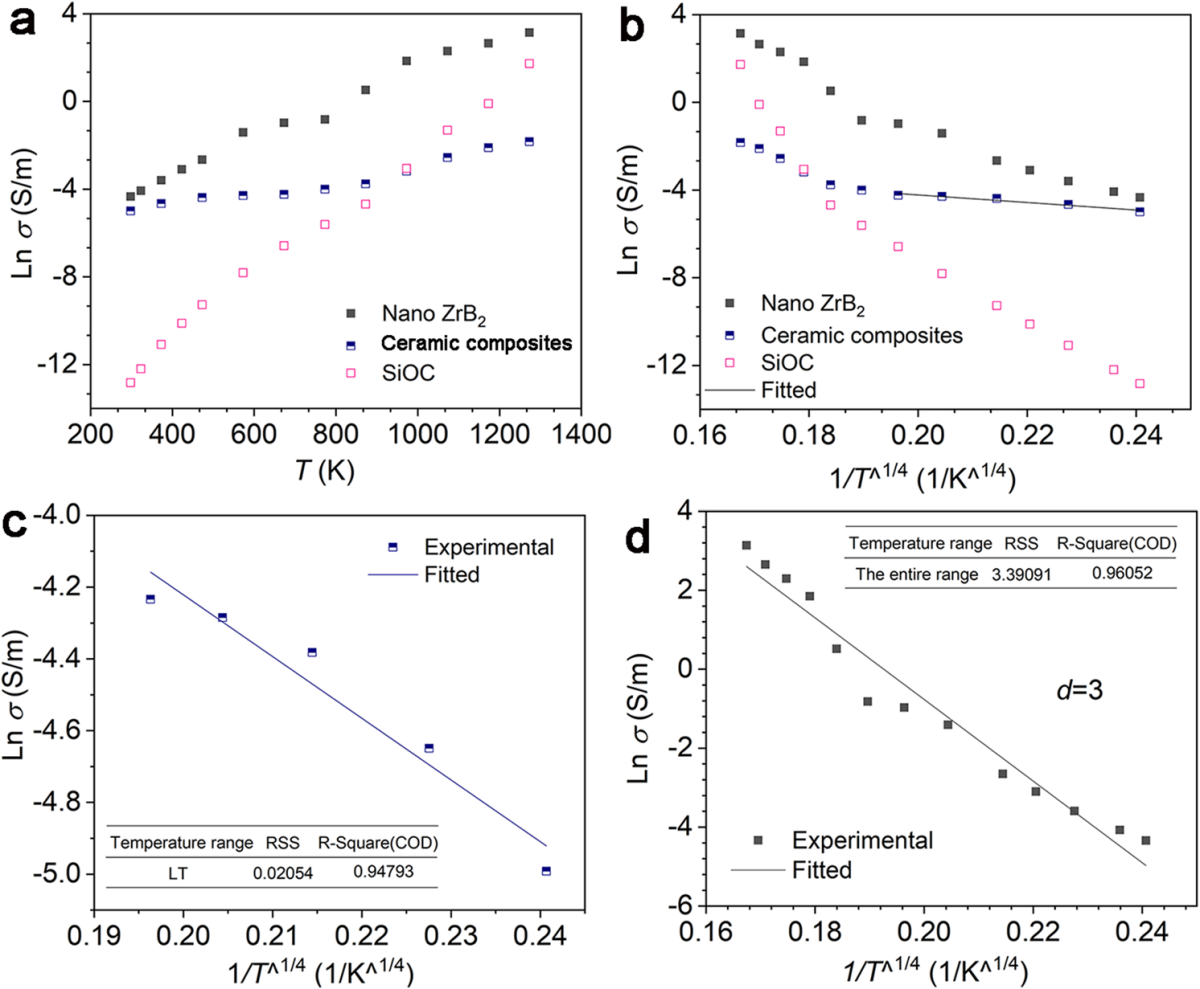
High-temperature DC conductivity and the corresponding fittings of different materials. (**a**) Ln σ ~ *T*, (**b**) Ln σ ~ *T*^^−1/4^, (**c**) the fitting for the ceramic composites, (**d**) the fitting for nano ZrB_2_.

Therefore, the high-temperature stability of the electrical property is important for the stabilization of the EM absorption of the ceramic composites at high temperature. In order to know the electron transport behavior of the ceramic composites, the high-temperature electrical conductivity was investigated at temperature up to 1000 °C. Theoretical models were used to analyze the experimental conductivity. The temperature-dependent conductivity for an amorphous semiconductor is usually described as^26^

9 $$\sigma ={\sigma }_{0}\mathrm{exp}\left(-\frac{A}{{T}^{\frac{1}{(1+d)}}}\right),$$

where *σ*, *T*, and *d* are the conductivity, temperature, and dimensionality, respectively. *σ*_0_ and *A* are constants. The plot reveals the Arrhenius equation, tunneling conduction, and three-dimensional hopping behavior when *d* is 0, 1, and 3, respectively. Figure 10 shows the high-temperature DC conductivity of the ceramic composites and nano ZrB_2_. From Fig. 10a, the conductivity of the three kinds of materials increased with the increase of temperature, revealing a semiconductor behavior. However, the nano ZrB_2_ reveals a higher DC conductivity than the ceramic composites as a function of temperature and the relationship between DC conductivity and temperature of these materials shows different variation trends. The slope of the temperature-dependent conductivity of the ceramic composites is the lowest among these materials, revealing the slow increase of the electrical conductivity. Figure 10b–d show the fitting of the conductivity for nano ZrB_2_ and the ceramic composites. The relationship can be fitted linearly using Eq. (9) when *d* is 3, which suggests that the conduction is predominantly controlled by the three-dimensional hopping mechanism. The DC conductivity of the ceramic composites in Fig. 10b reveals a two-stage change with temperature. For the ceramic composites, as the temperature increases, the conductivity shows a near flat variation up to high temperature (Fig. 10b). The stable temperature-dependent conductivity leads to the stable imaginary permittivity of the ceramic composites, which results in stable EM absorption at high temperatures.

**Figure 11:**
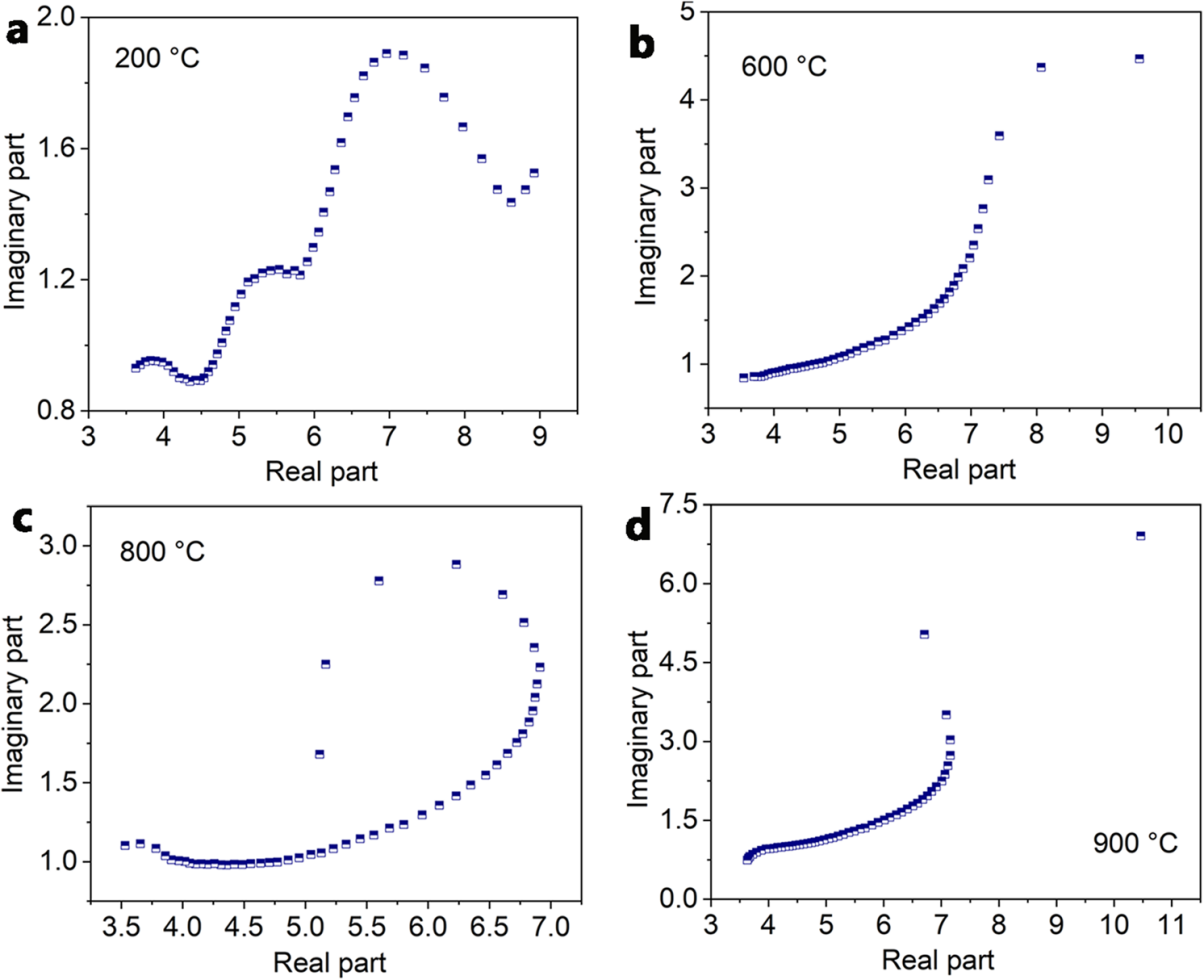
The plots of the real and imaginary permittivity of the ceramic composites at different temperatures.

Figure 11a–d shows the plots of the real and imaginary permittivity of the ceramic composites at different temperatures. These plots seem to contain semicircles (Cole–Cole semicircle) and each semicircle is related to one Debye relaxation process. The semicircles for the ceramic composites are normally attributed to the polarizations of different phases and the interfaces between them. In this work, the prescence of ZrB_2_/ZrO_2_ nanophases induces an extensive network of nano-interfaces within the composites. These nano-interfaces lead to strong polarization loss that contributes greatly to the attenuation of EM wave.

**Figure 12:**
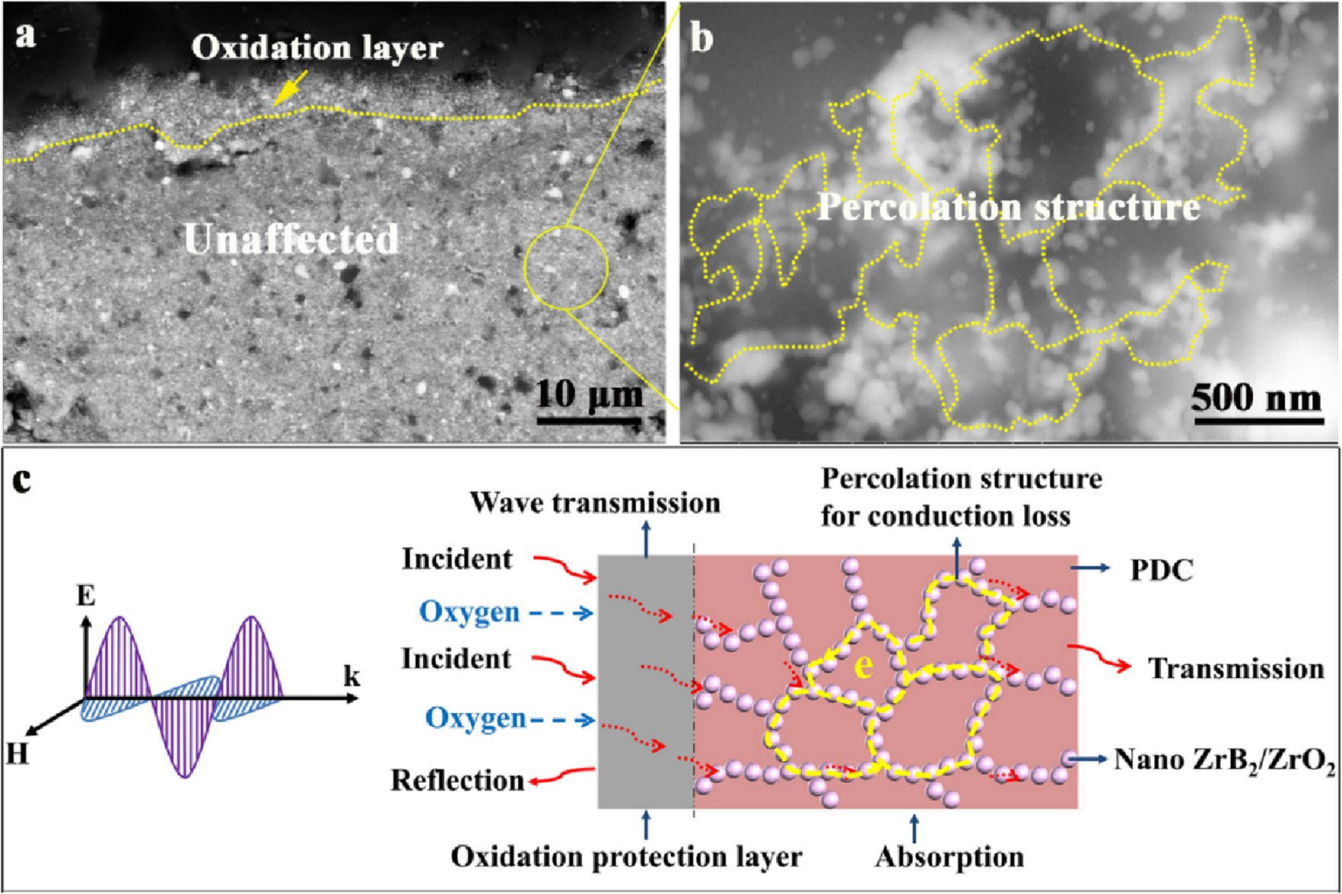
SEM images of the ceramic composites at 1000 °C in air showing the oxidation protection layer (**a**) and enlarged view revealing the percolation structures (**b**). Illustration of the EM absorption mechanism in high-temperature oxidation environment (**c**).

Good oxidation resistance is important for the ceramic composites to work at high temperature harsh environment. Figure 12a shows the SEM images of the ceramic composites processed at 1000 °C in air. This sample shows an oxidation protection layer, which is consistent with the observation in Fig. 2. In the enlarged view shown in Fig. 12b, percolated nanoparticles can be clearly observed within the SiOC matrix. These nanoparticles are the ZrB_2_/ZrO_2_ composite nanophases which serve as the EM absorbers for the ceramic composites. The percolated structures of these nanoparticles provide extensive conduction paths which enables the current to be induced through these paths and the electromagnetic energy can be dissipated by ohmic heating. Figure 12c illustrates the EM wave absorption mechanism in high-temperature oxidation environment with more details. The thin surface oxidation layer will provide oxidation protection for the bulk ceramic composites at high temperatures and allow the incidence of EM wave into the materials. The strong and stable electrical conduction loss provided by the ZrB_2_/ZrO_2_ composite nanophases induces the EM wave absorption for the ceramic composites in harsh environment.

Table 1 summarizes the microwave absorption bandwidth of typical ceramic-based composites as a function of temperature at different thicknesses reported in recent literature^5,6,10,22–32^. It can be seen that our ceramic composites show a stable and the widest bandwidth higher than 10 GHz from *RT* to 900 °C compared to other ceramic-based composites, such as oxide-based, PDC-based and other traditional ceramic based composites. This feature, combined with good thermomechanical properties, high-temperature stability and oxidation resistance, makes our ceramic composites ideal broadband EM absorbing materials for harsh environment applications.

**Table 1:** Microwave effective absorption bandwidth (EAB) of ceramic composite in this work and ceramic-based composites reported in literature.

Material	Temperature (°C)	Thickness (mm)	EAB (GHz)	Material	Temperature (°C)	Thickness (mm)	EAB (GHz)
Co_3_O_4_@rGO/SiO_2_^30^	80	1.94	4.20	SiC_f_/SiC-Al_2_O_3_^35^	25	3.00	2.70
	120		4.20		100		2.90
	160		4.20		200		3.10
	200		4.18		300		3.40
SiC_f_/mullite-SiO_2_^31^	200	2.50	3.37		400		4.10
	400		1.42		500		3.40
	600		0.00		600		2.20
C_f_/SiCnfs/Si_3_N_4_^8^	25	2.40	2.25		700		0.70
	400		2.42	Si_3_N_4_-SiC/SiO_2_^36^	25	3.30	4.20
	800		1.74		100		4.10
Ti_3_SiC_2_/codierite coating^32^	25	1.50	1.89		200		4.14
	100		1.91		300		4.16
	200		2.06		400		4.18
	300		2.02		500		4.16
	400		2.16		600		1.01
	500		2.46	Graphene@Fe_3_O_4_/SiBCN^13^	100	2.14	3.34
	600		2.75		200		3.50
	700		2.00		300		3.50
Fe-doped SiC/SiO_2_^33^	25	3.00	0.94		400		3.50
	100		0.59		500		3.41
	200		1.86		600		3.27
	300		3.28	RGO/Si_3_N_4_^37^	50	4.30	4.20
	400		3.82		100		4.20
	500		4.20		200		4.20
TiCnw/SiO_2_^34^	25	2.50	2.08		300		4.20
	100		2.35		400		4.20
	200		2.75		500		4.20
	300		2.99	Fe-SiBCN^28^	25	2.85	2.18
SiC_f_/SiC-SiCnw^9^	25	2.50	0.30		100		2.44
	200		2.86		200		2.78
	400		3.84		300		3.15
	600		2.90		400		3.59
					500		3.89
					600		4.20
BiFeO_3_^27^	27	1.80	0.69	Ni chains/SiO_2_^29^	50	1.80	1.36
	100		1.04		100		2.42
	150		1.38		150		2.42
	200		1.60		200		2.21
	300		2.29		250		1.65
	400		2.41		300		0.00
–				This work	25	3.00	13.50
					200		12.69
					400		12.69
					600		12.15
					800		11.88
					900		12.15
					1000		10.85

## Methods

### Fabrication of the ceramic composites

**Figure 13:**
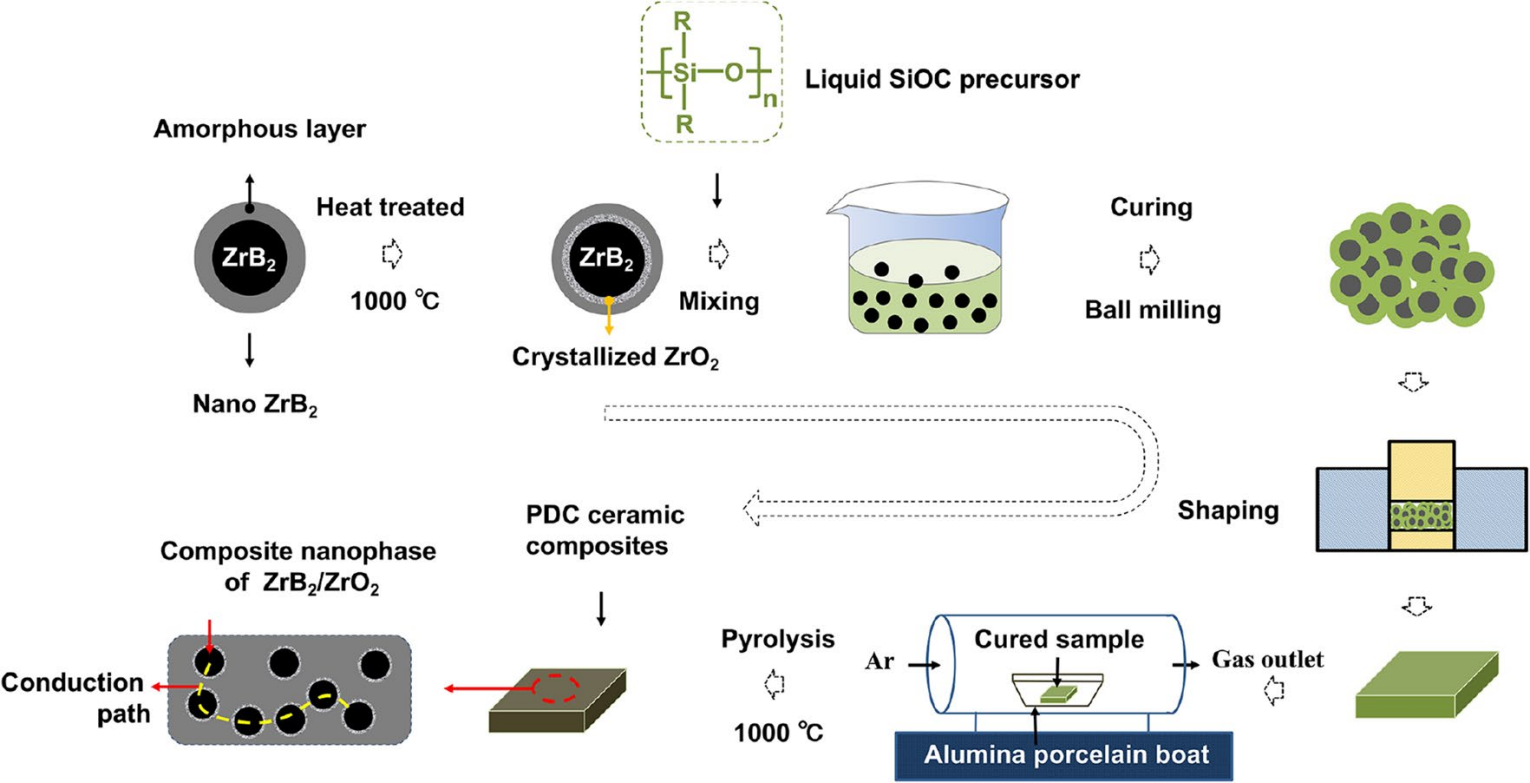
Illustration of the fabrication process of the ceramic composites.

Polycarbosiloxane (MS-154, Extreme Environment Materials Solutions, LLC) was used as the polymeric precursor for the matrix. The pyrolysis of the precursor resulted in the production of silicon oxycarbide (SiOC). ZrB_2_ nanopowders with a diameter ~ 43 nm (US Research Nanomaterials, lnc) were used as electrically conductive fillers and reinforcements for the PDCs. The ZrB_2_ nanopowders were subjected to heat treatment at 1000 °C for 3 h in Ar to alter the surface microstructure before being introduced into the PDC. Porior to the ceramic composite fabrication, 0.5 wt.% catalyzer (CLC-PB058, EEMS, USA) was added to the liquid-like polycarbosiloxane by electromagnetic stirring at *RT* until a homogeneous solution was obtained. Thereafter, 40 wt.% of the thermally processed ZrB_2_ nanopowders were added to 60 wt.% of the polycarbosiloxane/CLC-PB058 liquid and bleneded into a homogeneous mixture by mechanical stirring for about 30 min. The mixture was then cured in an oven at 120 °C overnight. A hard thermosetting product was obtained and it was high-energy ball milled into a uniform powder with size of ~ 1 µm. The powder was pressed into green body pellets of desired dimensions using different die sets. For pyrolysis, each pellet was placed in an alumina porcelain boat inside a tube furnace (GSL-1100X-LD, MTI Corporation, USA.). The samples were heated up to 1000 °C with a rate of 2 °C/min and held for 2 h in Ar and then cooled down to *RT* with a cooling rate of 2 °C/min. The detailed preparation process of the nanocomposites is illustrated in Fig. 13. An external standard and Rietveld methods were used to quantify the phase fractions in the ceramic composites using the XRD curve of the un-oxidized sample shown in Fig. 4i ^38,39^. The results are shown in Table 2. The pure SiOC ceramic was also prepared using the same route for comparison.

**Table 2:** Phase fractions in the ceramic composites.

Phase	Weight percentage (%)
ZrB_2_	36.4
ZrO_2_	3.6
SiOC	60.0

### Characterization

Field emission scanning electron microscope coupled with Energy Dispersive X-Ray Spectroscopy (SEM, FEI Verios 460L, USA) and transmission electron microscopy (TEM) were used to characterize the micro- and nanostructures of the materials. TEM investigation was performed using a Talos F200X G2 (USA) microscope operated at 200 keV. The phase compositions were investigated by X-ray Diffraction analysis (Rigaku SmartLab, Tokyo, Japan) at *RT*. High-temperature in-situ XRD characterizations of the nanocomposites were conducted at 25–1150 °C in air, with a heating rate of 10 °C/min.

**Figure 14:**
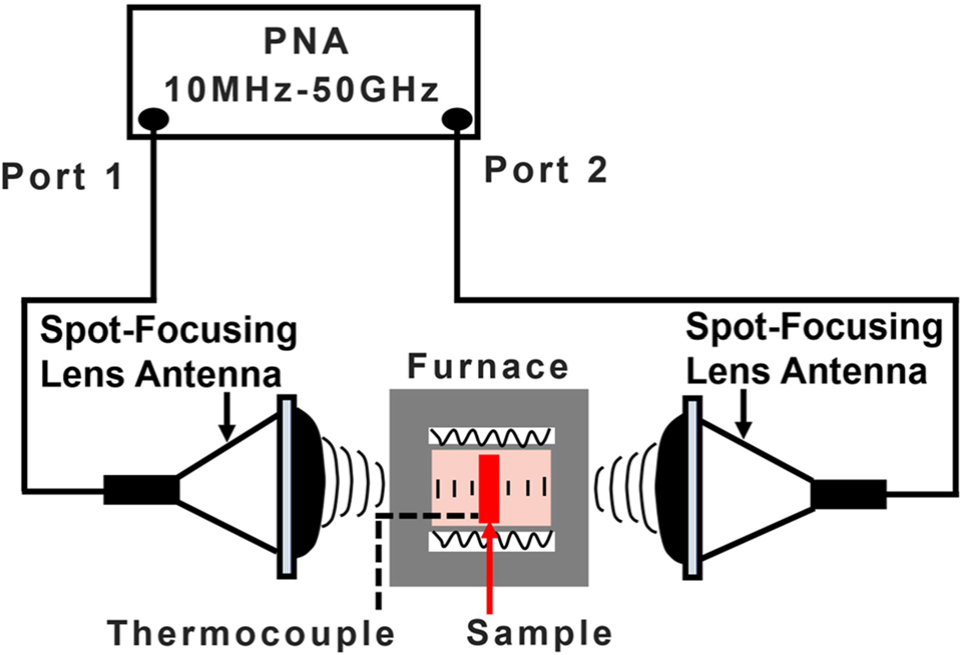
Illustration of the measurement device for high-temperature microwave absorption^40,41^.

The high-temperature relative complex permittivity of the ceramics was measured through the free space method. The measurement setup consisted of a vector network analyzer (Keysight, N5225A PNA, 10 MHz to 50 GHz), transmit and receive antennas (spot-focusing lens antennas), and a furnace (Fig. 14). The sample size for the free space test was 40 mm by 40 mm by 2.5 mm. A TRL (through, reflect, line) calibration method was used to calibrate the measurement. For the through calibration, the distance between the two antennas was set to be equal to twice the focal distance. The reflect standards are achieved by placing a metal plate at the focal planes of the two antenna. For the line calibration, the focal planes of the two antennas were separated by a distance equal to a quarter of the wavelength at the center of the frequency band. The samples were heated and kept at the desiginated temperatures (200, 400, 600, 800, 900, and 1000 °C) for 1 h during the measurements. Their microwave scattering parameters at these temperatures were directly measured by the PNA in the frequency range of 26.5–40 GHz (Ka-band). Permittivity values of the samples were calculated according to the Nicolson–Ross–Weir (NRW) algorithm. The sample surface was polished before measurement.

High-temperature direct current (DC) conductivity was measured by the I–V curve on Keithley 2000 multimeter (Tektronix, Inc. Beaverton, USA). Carbon paint (SPI, West Chester, Pennsylvania, USA) was applied on the sample surface as electrodes. Platinum wires with a diameter of 0.25 mm were used as conducting wires and connected to the samples. The platinum wires were loaded in a two-bore Al_2_O_3_ tube to prevent short circuit during the measurement. Samples were heated at a rate of 5 °C/min in the hot zone of a furnace in Ar. The resistance was recorded at temperatures up to 1000 °C with an interval of 100 °C. Samples were kept for 5–10 min at each temperature point before measurement to avoid temperature fluctuation. The resistance of the platinum wire was also measured at each temperature point. The ultimate sample resistance was the value excluding that of the platinum wire. The conductivity (*σ*) was calculated according to the following equation.

10 $$\sigma = \frac{l}{RS},$$

where *l*, *R*, *S* are the thickness, resistance, and sectional area of the samples. The reported conductivity was the average value from three samples of each type.

For the DMA test (TA Instruments Discovery DMA 850 Dynamic Mechanical Analyzer with GCA tank), the temperature sweep starts from *RT* to 350 °C at 1 °C/min to avoid thermal shock in the sample and allows the sample to cool completely to *RT* before performing another test at a different frequency. For these tests, 1 N preload, 14 µm oscillation amplitude (~ 0.015% strain), and 175% force track (ratio between dynamic and static forces) were used. The temperature sweeps were performed at 0.1 Hz, 1 Hz, 10 Hz, and 100 Hz loading frequencies to help assess if the storage modulus changed with the dynamic loading rate.

The ceramic composites were exposed to a mixture of Ar and water vapor (Ar: H_2_O molar ratio was of about 5:1) flowing at 100 cm^3^/min from *RT* to 500 °C for the water vapor oxidation test. Before the test, the samples were dehumidified in an oven at 120 °C for 2 h. In such a water–vapor-containing environment, the primary oxidant is water vapor. The sample mass before and after the water vapor oxidation test was measured to calculate the mass change of the ceramic composites.

Thermal shock test of the ceramic composites was conducted by water quenching at *RT*. The samples were completely dried in an oven at 100 °C overnight before the experiment. The thermal shock behavior of the ceramic composites was evaluated as a function of quenching temperature and quenching cycles. The samples were heated at a rate of 5 °C/min to a preset temperature (800 °C) in a tube furnace (Carbolite gero 30-3000C HTRH 18/100/600, Sheffield, United Kingdom) in air and held for 10 min. Then, the heated samples were dropped by free fall into the water bath at *RT*. Each sample was tested 5 times.

The ceramic composites were also subjected to the jet flow environment to test the structural stability under thermal impact. The jet facility at Florida Agricultural and Mechanical University—Florida State University (FAMU-FSU) college of engineering utilizes high-pressure (3500 kPa) compressed air to generate high Mach number jets. The compressed air was heated using an electric heater at a stagnation temperature of 254 °C. The sample size for the test is 7.112 mm × 3.556 mm × 3.018 mm.

## Conclusions

This paper reports a type of ceramic composite with stable microwave absorption from *RT* to 900 °C, which is made of polymer-derived SiOC as the matrix and core–shell nanophase structures of ZrB_2_/ZrO_2_ as the microwave absorbers. Crystallized ZrO_2_ acts as the interface layer between the SiOC and ZrB_2_. Electrical, dielectric, and microwave absorption properties of the ceramic composites were systemically investigated at different temperatures. The ceramic composites show a significantly wide microwave absorption bandwidth especially between 28 and 40 GHz from *RT* to 900 °C. The stable EM absorption properties of the ceramic composites at high temperatures are attributed to the core–shell nanophase structure of ZrB_2_/ZrO_2_, which induces the stable dielectric and electrical properties of the ceramic composites. Crystallized t-ZrO_2_ also increases the nano-interfaces in the composites, enhancing the polarization loss of electromagnetic waves. The DC conductivity of the ceramic composites shows a stable temperature-dependent trend because of the existence of the t-ZrO_2_ interface. Results of the thermomechanical analysis, jet flow, thermal shock, and water vapor tests show that the ceramic composites have good harsh environmental stability. The excellent microwave absorption properties make the presented ceramic composites an ideal microwave absorbing material for high-temperature harsh environment applications.

## Data Availability

The datasets used and/or analyzed during the current study are available from the corresponding author on reasonable request.
